# Perceptions and Practices towards Anthrax in Selected Agricultural Communities in Arua District, Uganda

**DOI:** 10.1155/2020/9083615

**Published:** 2020-09-16

**Authors:** Joseph M. Kungu, Peninah Nsamba, Alfred Wejuli, John D. Kabasa, William Bazeyo

**Affiliations:** ^1^College of Veterinary Medicine, Animal Resources and Biosecurity, Makerere University, Kampala, Uganda; ^2^Ministry of Agriculture Animal Industry and Fisheries, Entebbe, Uganda; ^3^College of Health Sciences, Makerere University, Kampala, Uganda

## Abstract

**Background:**

Anthrax is globally recognized as an important public health and economic challenge in many agricultural communities. A cross-sectional study was conducted in three subcounties in Arua district to assess the community's awareness, cultural norm, and practices regarding anthrax. This followed a report of active cases of human cutaneous anthrax in the district.

**Methods:**

The study was conducted in subcounties of Pawor, Rigbo, and Rhino Camp, Arua district, using focus group discussion.

**Results:**

The affected communities had limited knowledge about anthrax, especially its clinical manifestation and modes of transmission both in humans and animals. The community also had no knowledge of the anthrax vaccine or treatment and where they can be accessed from. Poor practices associated with anthrax outbreaks included poor disposal of carcasses and ruminal wastes, occupational hazards (butchers, slaughter men, and herdsmen), consumption of meat from infected animals, communal herding, and cultural norms encouraging consumption of dead animals.

**Conclusion:**

This study shows that there is a knowledge gap about anthrax among the people in the affected communities. Key drivers for the anthrax outbreak such as poor cultural beliefs and practices and wildlife-livestock-human interactions were observed in all the three subcounties studied. All these findings could imply a high risk of outbreak of anthrax in Arua and Ugandan agricultural communities where the public health programs are less standardized and less effective.

## 1. Introduction

Anthrax is a bacterial disease of public health and economic importance endemic in many agricultural parts of the world [1]. Anthrax is caused by the Gram-positive *Bacillus anthracis*, which affects herbivorous animals (wild and domestic) and humans [2–5]. Livestock get infected through ingestion or inhalation of spores from contaminated soil, water, or plants with the clinical course of the infection ranging from peracute to chronic [1, 6]. Human beings are exposed through contact with infected or dead animals or their products especially through abrasions and inhalation [1]. Anthrax in humans manifests in three forms (cutaneous, gastrointestinal, and respiratory), the most common being the cutaneous form characterized by blisters or bumps that may itch on the face, neck, arm or hands, swelling around the sore, and a painless skin sore with a black center [7, 8].

A number of factors such as changing rainfall patterns, soil disturbance, increased animal and human populations, and poor grazing systems and human behavior have been reported to be associated with outbreaks of anthrax [7]. Interaction of wildlife with livestock and humans has also been reported as a key predisposing factor of anthrax among humans and livestock. The disease usually reoccurs in areas where there has been a previous outbreak, making vaccination of the recommended form of control [1, 9].

Although the true incidence of anthrax globally is not known due to poor diagnosis and reporting, the disease has been reported from all continents, especially in agricultural communities with neutral or alkaline, calcareous soils [1, 10]. In Africa, anthrax remains a major problem except in South Africa where it continues to be at a low sporadic incidence probably as a result of the livestock owners taking the central role of control [11]. Continuous sporadic outbreaks of the disease have been reported in a number of countries in the sub-Saharan Africa including Uganda in the recent years [3]. According to the Ministry of Health of the Republic of Uganda, two human cases had been confirmed to have anthrax infection in Arua district in April 2018. These cases had emanated from a community where the infection had previously been experienced in 2017 among both humans and livestock.

Since previous studies have shown that poor perceptions, cultural norms, beliefs, and practices of local communities play key roles in the persistence of anthrax outbreaks, it was in the interest of this study to investigate this phenomenon with regard to the outbreak in Arua district [9]. The findings obtained were expected to provide a basis for combating the recurrent outbreaks.

## 2. Methods

### 2.1. Study Sites

A cross-sectional study was conducted in Pawor, Rhino Camp, and Rigbo subcounties, Arua district, from 1^st^ to 30^th^ of May 2018. Since February 2017, a total of 155 cattle and one person had been reported to have died from suspected anthrax infection in the three subcounties [2].

**Figure 1:**
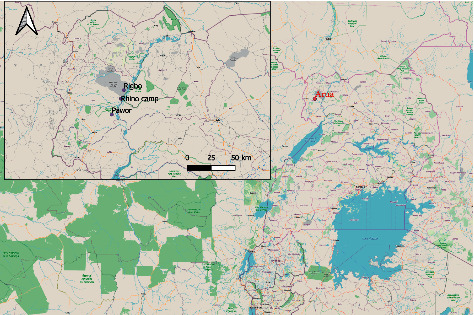
Location of study sites (source: QGIS maps).

Arua district is located in West Nile region, Uganda. It has an area of 3,236.4 km^2^ (1,249.6 sq mi) comprising 28 subcounties. Arua district is bordered by Yumbe district to the north, Adjumani district to the northeast, Amuru district to the east, Nebbi district to the southeast, Zombo district to the southwest, the Democratic Republic of Congo to the west, and Maracha district to the northwest as shown in Figure 1. Arua district has a total population of 840,900 people, with farming as the major economic activity where up to 144,090 cattle, 314,832 goats, and 54,693 sheep, respectively, are kept [12].

### 2.2. Study Design

Three subcounties, namely Rhino Camp, Rigbo, and Pawor, were purposively selected based on reports of previously confirmed outbreaks of anthrax. Participants in the respective study subcounties were recruited with help from village opinion leaders who acted as guides. The inclusion criteria for study participants were (1) previous exposure to sick or dead animals suspected to have been infected with anthrax, (2) previous contact with raw infected meat, and (3) consumption of boiled/roasted meat suspected to be infected. An animal case of anthrax was defined based on the Food and Agricultural Organization of the United Nations (FAO), and a human case was defined based on the World Health Organization [1, 13].

### 2.3. Data Collection

We conducted 12 focus group discussions (FGDs) in the study communities to gather detailed qualitative data on perceptions and practices associated with anthrax, using an open-ended question checklist. Each focus group constituted 10–12 participants as summarized in Table 1. The selection of participants ensured homogenous involvement of both men and women with suspected exposure to anthrax. Their participation was solely voluntary, and their responses were treated with confidentiality. The group discussions were conducted by two moderators (JMK and AW) with aid of a recording audio device for later transcription.

### 2.4. Data Analysis

The recorded audio files of the focus group discussions were translated into English and later transcribed into Ms Word files by JMK and AW. The transcribed data were then transferred to QDA Minor Lite (version1.4.1) for coding into themes based on the objectives of the study (coded data attached as a supplementary file) [14].

Summarized narratives under each theme were also generated. Key illustrative quotations of the FGDs have also been noted under the themes. The themes included demographic and socioeconomic characteristics, community awareness of anthrax, and practices associated with anthrax outbreaks.

## 3. Results

### 3.1. Demographic and Socioeconomic Characteristics

Participants of the FGDs were within the age bracket 18–70 years with 85% of these being <50 years and mostly males (65%). The main source of livelihood in the study communities was noted to be subsistence farming (>90%), although other activities such as fishing, charcoal burning, casual labor, papyrus cutting, and brewing local beer were performed to supplement income.

Up to 70% of the participants had attained a primary level of formal education, 25% secondary, and few (5%) had not undergone formal education.

**Figure 2:**
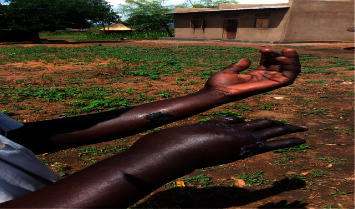
Active case with cutaneous anthrax in Pulwal village (photograph by JMK).

Two suspected cutaneous anthrax cases, male by gender, were observed in Pulwal village in Pawor subcounty in Arua district where both suspects had been involved in slaughtering of dead animals. The suspected cases had developed skin lesions characterized by itching of the affected area followed by papular lesions with a vesicular stage for 2–6 days, eventually developing into depressed black eschar with edema (Figure 2). No animals were observed to have evidence of clinical manifestation of the disease at the time of the study.

### 3.2. Awareness of Participants about Anthrax

Community members had limited knowledge about anthrax especially its manifestation and modes of transmission both in humans and animals. However, following an illustration of the clinical signs of the disease using pictures of typical cases, it was noticed that some community members had heard and or seen people and animals with these signs, even though they mistook them for other conditions. For instance, those who had experienced clinical manifestation of anthrax considered it to be malaria, skin abrasions, abdominal pain, headache, or witchcraft. A participant said “our neighbor lost 12 goats within three days and we believed it was a malicious act of witchcraft. The dead goats looked exactly like what is in the pictures,” female FGD, Ocea.

Another participant also said *“*The way you have explained to us about the signs of this condition in humans, it is possible we have been experiencing it but we treat it like malaria, stomach upset, and headache. We usually go to the clinics and buy some tablets, and it will clear,” female FGD, Parabok. Anthrax was not mentioned among the important diseases of livestock during the discussions. The community also had no knowledge of the anthrax vaccine or treatment and where they can be accessed from.  Table 2 provides a summary of key quotes of the focus group participants with regard to awareness of anthrax.

### 3.3. Practices Associated with Outbreak of Anthrax

The practices that could be an encouraging outbreak of anthrax in the three subcounties included poor disposal of dead animals, occupational risks, consumption of meat, and cultural practices. Disposal of dead livestock was considered a taboo, which would result into the remaining herd being wiped out and wealth being lost. It was also observed that all those who had been reported to have anthrax had been involved in slaughter and eating of a dead cow that had died with clinical signs of anthrax. Quotes of participants are summarized under three themes (Table 3).

## 4. Discussion

For successful control and possible eradication of diseases of public health importance such as anthrax, community awareness about the condition is primarily important [15, 16]. This study assessed the perceptions of affected agricultural communities in Arua district regarding anthrax and practices that could facilitate its spread. There was a low level of awareness about anthrax and its importance as a zoonosis in the studied communities. This report is contrary to a study in agricultural communities in Zambia and Zimbabwe where the knowledge about anthrax was observed to be considerably high [7, 16]. This difference could have been because anthrax outbreaks in the areas studied in the two countries were rampant, making the high-risk communities vigilant [9]. Despite the limited knowledge about anthrax, it was noted that the zoonotic condition could be occurring although its manifestations are mistaken to be symptoms of endemic diseases such as malaria, typhoid, and diarrhea in humans and tick-borne diseases in livestock. Although the active cases reported having received antibiotic treatment for their persistent wounds, participants in the discussion generally agreed that they did not know how to treat or prevent the disease in both humans and livestock. This study shows evidence of an existing knowledge gap among the affected communities that needs to be urgently addressed to prevent impending recurrence of anthrax.

Findings indicated that the two active cases identified in this study had had direct contact with a dead animal during dressing and distribution of the carcass, which could have exposed them to spores resulting in localized skin lesions. They also admitted to have eaten the meat after boiling or roasting, a practice which could have probably limited manifestation of gastrointestinal form of anthrax [1]. Similar findings were reported in Nakuru, Kenya, whereby identified active cases of cutaneous and gastrointestinal anthrax were traced to previous contact with infected cattle [5]. A study in Western and Muchinga provinces, Zambia also reported that humans with anthrax had eaten infected beef and hippo (*hippopotamus amphibius*) meat [7]. In northern Tanzania, infection of humans with cutaneous anthrax following their involvement in skinning and butchering of infected animal carcasses was reported in a case control study [17]. Since there is evidence of outbreak of anthrax in the community, there is need for the people to be sensitized about the condition, especially by targeting the high-risk groups who are involved in herding and slaughtering of livestock.

Cultural norms such as “burying a dead animal meant burying wealth” and the practice of esteeming meat as “a treasure that cannot be wasted” were noted in this study. These beliefs and practices were similarly reported in a study by Sitali et al., as important drivers of persistent outbreaks of anthrax [9]. The beliefs could be facilitated by lack of knowledge of the community about implications of an anthrax outbreak and limited access to animal source foods due to high poverty levels. The dead animals provide a cheaper source of meat that could be accessed at a minimal cost, barter trade, or on loan. Other practices observed in this study, such as herding livestock communally, whereby animals share common grazing and watering points, could be facilitating the spread of anthrax. Communal livestock herding also complicates decision making when treatment or vaccination of the animals is envisaged [8, 18].

Human-livestock interaction with wildlife was observed to occur in communities neighboring Ajai wildlife reserve and could increase risk of spread of anthrax from possible wildlife reservoirs of the infection [19]. There is need for the communities to minimise interaction with the wildlife reserve.

## 5. Study Limitation

This study was not able to explain possible occurrence of the inhalation and gastrointestinal forms of anthrax in humans. The possibility of subclinical disease among livestock in the community was also not ascertained.

## 6. Conclusion

This study shows that there is a knowledge gap about anthrax among the people in the affected communities. Key drivers for the anthrax outbreak such as poor cultural beliefs and practices and wildlife-livestock-human interactions were observed in all the three subcounties studied. All these findings could imply a high risk of outbreak of anthrax in Arua and Ugandan agricultural communities where the public health programs are less standardized and less effective.

Basing on these findings, the people need to be sensitized about anthrax, clinical manifestation, transmission, and how it can be prevented. To successfully combat the outbreak of anthrax in Arua district, active involvement of medical, veterinary, wildlife personnel, and farmers should be envisaged, with sensitization of affected communities about anthrax as a beginning step.

## Figures and Tables

**Table 1:** Qualitative data collection in selected subcounties, Arua district.

Subcounty	Villages	Number of FGDs
Rhino camp	Ombeniva, Ndara, and Janduwa	6
Pawor	Pulwal and Parabok	4
Rigbo	Ocea	2

**Table 2:** Quotes of participants on awareness about anthrax.

Theme	Quote
Common diseases that affect humans in the community	“Our biggest challenge has always been fever, malaria, cough, diarrhea, and typhoid. We are grateful to government that they have supplied mosquito nets, carried out deworming, and vaccination of children and pregnant mothers to reduce the burden of these diseases in our community. On some occasions, we experience symptoms similar to those shown in the pictures, but they clear by themselves,” female FGD, Parabok.
Common diseases that affect animals in the community	“Livestock suffer a lot with diseases of ticks, tsetse flies, and worms. These diseases are so common especially during rainy season. After seeing pictures of cases with anthrax, we realize it could be occurring, but we have not been keen to know,” male FGD, Janduwa.
Heard of anthrax	“Who would have told us about this anthrax, if it was not for you facilitators to sensitize us? There is a rumor of a strange skin disease among people in Pulwal whom they say ate dead carcasses”, male FGD, Ocea. “We were sensitized last month by the health workers during our antenatal visits to the health center about a strange disease, which is spreading in our community after eating dead meat,” female FGD, Ombeniva.
Transmission and clinical signs	“Sudden death occurred of 5 cattle in our village herd, and we decided to dress the carcasses for consumption. Two of the six of us suffered skin lesions. Several attempts to treat myself were in vain, but that very week, a team from Arua Hospital picked samples from us and reported later to us that I have anthrax,” male case, Pulwal. “One month ago, our neighbor lost 12 goats within three days, and we suspected malice of a bad neighbor using witchcraft. It is now clear after seeing these pictures and explanations of the facilitators that it could have been anthrax,” female FGD, Ocea.
Control of anthrax	“When skin lesions appeared and persisted, I visited nearby clinics and bought some Ampiclox capsules but I was unable to buy a full dose because I did not have money. However, we did not do anything for the livestock since they were not dying anymore,” male case, Pulwal.

**Table 3:** Quotes of participants on practices associated with anthrax outbreak.

Theme	Quote
Occupational risks	“Since this condition seems to come from animals, herdsmen and people who slaughter livestock are in danger of getting the disease,” male FGD, Ndara. “I should have acquired the disease when I participated in dressing a dead cow, probably through bruises. I had ignored the swelling on my hand but realized it had persisted and become wider. I think this meat was safe to eat since my family members whom I shared the meat with did not fall sick,” male case, Pulwal.
Proximity to wildlife reserve	“Many of us in the community enter Ajai game reserve to graze livestock, harvest firewood, and hunt game meat, but nobody has fallen sick or died because of that,” male FGD, Parabok.
Cultural practices, norms, and beliefs	“To minimise costs and theft, the livestock in our communities are kept, grazed, and watered communally,” male FGD, Rhino Camp. “It is possibly true that we could have stopped anthrax from spreading by burying the dead cow. However, in our culture, it is a very big taboo to bury dead livestock because you will have buried wealth and your herd can never multiply,” male FGD, Pulwal. “It is usually difficult to rule out malicious tendencies due to witch craft when a person loses many livestock within a short period,” female FGD, Ocea.

## Data Availability

No data were used to support this study.

## Abbreviations

FGD: Focus group discussion.

## Supplementary Materials

Supplementary Materials The data which were coded in QDA Minor Lite software have been provided as supplementary material.
