# Supplementary material for: Perceptions and Practices towards Anthrax in Selected Agricultural Communities in Arua District, Uganda
Source: J Trop Med. 2020 Sep 16;2020:9083615. doi: 10.1155/2020/9083615 (PMC7519178; doi:10.1155/2020/9083615)
Supplement: Supplementary Materials — The data which were coded in QDA Minor Lite software have been provided as supplementary material. [file 9083615.f1.pdf]

Category	Code	Case
Demographic characteristics	Age range of respondents	Janduwa data
Demographic characteristics	Sex of respondents	Janduwa data
Demographic characteristics	Economic activities	Janduwa data
Awareness of anthrax	Common diseases of humans	Janduwa data
Awareness of anthrax	Common diseases of livestock	Janduwa data
Awareness of anthrax	Heard of anthrax	Janduwa data
Cultural norms, beliefs and practices associated with anthrax	Occupational risks	Janduwa data
Cultural norms, beliefs and practices associated with anthrax	Cultural norms and beliefs	Janduwa data
Demographic characteristics	Age range of respondents	Ndara data
Demographic characteristics	Sex of respondents	Ndara data
Demographic characteristics	Economic activities	Ndara data
Demographic characteristics	Economic activities	Ndara data
Awareness of anthrax	Common diseases of humans	Ndara data
Awareness of anthrax	Common diseases of livestock	Ndara data
Awareness of anthrax	Heard of anthrax	Ndara data
Awareness of anthrax	Heard of anthrax	Ndara data
Awareness of anthrax	Transmission and clinical signs of anthrax	Ndara data
Awareness of anthrax	Transmission and clinical signs of anthrax	Ndara data
Cultural norms, beliefs and practices associated with anthrax	Cultural norms and beliefs	Ndara data
Cultural norms, beliefs and practices associated with anthrax	Cultural norms and beliefs	Ndara data
Demographic characteristics	Age range of respondents	Ocea data
Demographic characteristics	Sex of respondents	Ocea data
Demographic characteristics	Economic activities	Ocea data
Demographic characteristics	Economic activities	Ocea data
Awareness of anthrax	Heard of anthrax	Ocea data
Awareness of anthrax	Transmission and clinical signs of anthrax	Ocea data
Awareness of anthrax	Transmission and clinical signs of anthrax	Ocea data
Cultural norms, beliefs and practices associated with anthrax	Cultural norms and beliefs	Ocea data
Cultural norms, beliefs and practices associated with anthrax	Cultural norms and beliefs	Ocea data
Cultural norms, beliefs and practices associated with anthrax	Cultural norms and beliefs	Ocea data
Cultural norms, beliefs and practices associated with anthrax	Cultural norms and beliefs	Ocea data
Demographic characteristics	Age range of respondents	Ombeniva data.docx
Demographic characteristics	Sex of respondents	Ombeniva data.docx
Demographic characteristics	Economic activities	Ombeniva data.docx
Awareness of anthrax	Common diseases of humans	Ombeniva data.docx
Awareness of anthrax	Common diseases of livestock	Ombeniva data.docx
Awareness of anthrax	Heard of anthrax	Ombeniva data.docx
Awareness of anthrax	Transmission and clinical signs of anthrax	Ombeniva data.docx
Cultural norms, beliefs and practices associated with anthrax	Cultural norms and beliefs	Ombeniva data.docx
Demographic characteristics	Age range of respondents	Parabok data.docx
Demographic characteristics	Sex of respondents	Parabok data.docx

Category	Code	Case
Demographic characteristics	Economic activities	Parabok data.docx
Awareness of anthrax	Common diseases of humans	Parabok data.docx
Awareness of anthrax	Common diseases of livestock	Parabok data.docx
Awareness of anthrax	Common diseases of livestock	Parabok data.docx
Awareness of anthrax	Heard of anthrax	Parabok data.docx
Awareness of anthrax	Transmission and clinical signs of anthrax	Parabok data.docx
Cultural norms, beliefs and practices associated with anthrax	Cultural norms and beliefs	Parabok data.docx
Cultural norms, beliefs and practices associated with anthrax	Proximity to wildlife reserve	Parabok data.docx
Cultural norms, beliefs and practices associated with anthrax	Cultural norms and beliefs	Parabok data.docx
Demographic characteristics	Age range of respondents	Pulwal data
Demographic characteristics	Sex of respondents	Pulwal data
Demographic characteristics	Economic activities	Pulwal data
Demographic characteristics	Presence of active cases	Pulwal data
Awareness of anthrax	Common diseases of humans	Pulwal data
Awareness of anthrax	Common diseases of livestock	Pulwal data
Awareness of anthrax	Transmission and clinical signs of anthrax	Pulwal data
Awareness of anthrax	Heard of anthrax	Pulwal data
Awareness of anthrax	Control of anthrax	Pulwal data
Cultural norms, beliefs and practices associated with anthrax	Occupational risks	Pulwal data
Cultural norms, beliefs and practices associated with anthrax	Cultural norms and beliefs	Pulwal data

Text	Words	% Words
Participants of the FGDs were within the age bracket 18-60 year	19	0.70%
more females (55%)	3	0.10%
subsistence farming, although other activities such as fishin	10	0.40%
we experience symptoms similar to those shown in the pict	15	0.60%
L ivestock suffer a lot with diseases of ticks, tsetse flies ar	44	1.60%
a strange disease which is spreading in our community afte	13	0.50%
poor disposal of dead animals, occupational risks, consumption	10	0.40%
Disposal of dead livestock is a taboo which would result into the	21	0.80%
he age bracket 18-65 years	6	0.20%
50% male and 50% female.	5	0.20%
subsistence farming	2	0.10%
charcoal burning, casual labor, papyrus cutting, brewing loca	9	0.40%
fever, malaria, cough, diarrhea,	4	0.20%
diseases of ticks, tsetse flies and worms.	7	0.30%
a strange disease which is spreading in our community afte	13	0.50%
We heard that people are sick in Pulwal with a sickness of anima	13	0.50%
a sickness of animals	4	0.20%
Since this condition seems to come from animals, herdsmen and	21	0.80%
livestock in our communities are kept, grazed and watered	10	0.40%
a very big taboo to bury dead livestock because you will have	20	0.80%
within the age bracket 18-66 years	7	0.40%
with more females (55%)	4	0.20%
subsistence farming	2	0.10%
casual labor, papyrus cutting, brewing local beer	7	0.40%
Who would have informed us of this "anthrax" if it was not for yo	39	2.00%
animals are dying suddenly in our village	7	0.40%
One month ago, our neighbor lost 12 goats within three days and	40	2.00%
One month ago, our neighbor lost 12 goats within three days and	21	1.10%
a taboo which would result into the remaining herd being wipe	16	0.80%
ommunities are kept, grazed and watered communally	7	0.40%
ur culture it is a very big taboo to bury dead livestock because	18	0.90%
Participants of the FGDs were within the age bracket 18-70 year	12	0.50%
femala and males equivalent proportion	5	0.20%
crop farming , livestock keeping, fishing, charcoal burning, c	9	0.30%
fever, malaria, cough,	3	0.10%
ticks, tsetse flies	3	0.10%
We were sensitized last month by the health workers durin	31	1.20%
spreading in our community after eating dead meat	8	0.30%
It is a bad omen to bury dead livestock. You leave people to e	15	0.60%
within the age bracket 18-70 years	7	0.30%
mostly males (65%	3	0.10%

Text	Words	% Words
grow crops and keep cattle, pigs, sheep and goats, fishing, c	14	0.60%
ur biggest challenge has always been fever, malaria, cough, dia	61	2.70%
diseases of ticks, blindness, tsetse flies, lumpy skin disease a	11	0.50%
diseases of ticks, blindness, tsetse flies, lumpy skin disease a	11	0.50%
Heard of sudden death of cattle in neighboring Pulwal	9	0.40%
sudden death of cattle	4	0.20%
Burning or burying of dead livestock is not allowed in our culture	12	0.50%
Many of us in the community enter Ajia game reserve to graze liv	29	1.30%
the livestock in our communities are kept, grazed and wat	11	0.50%
e FGDs were within the age bracket 18-70 years	10	0.50%
mostly females (55%).	3	0.20%
subsistence farming (>90%), although other activities such a	18	0.90%
Two suspected cutaneous anthrax cases, male by gender were	65	3.30%
malaria, cough, diarrhea, and typhoid	5	0.30%
diseases of ticks, tsetse flies	5	0.30%
Sudden death occurred of 5 cattle in our village herd and we dec	58	3.00%
Two of the six of us suffered skin lesions. Several attempts to	39	2.00%
When skin lesions appeared and persisted, I visited nearby clinic	46	2.40%
Since this condition seems to come from animals, herdsmen and	78	4.00%
It is possibly true that we could have stopped anthrax from sprea	43	2.20%
